# ARE INEQUALITIES IN HEART DISEASE CHARACTERIZED BY INTERGENERATIONAL EDUCATIONAL ATTAINMENT?

**DOI:** 10.1093/geroni/igad104.3074

**Published:** 2023-12-21

**Authors:** Blakelee Kemp, Carli Rush, Patricia Morton

**Affiliations:** University of Nebraska, Lincoln, Nebraska, United States; University of Nebraska, Lincoln, Nebraska, United States; Wayne State, Detroit, Michigan, United States

## Abstract

Intergenerational patterns in educational attainment are associated with later-life heart health. Yet, whether the association between intergenerational education patterns and adult heart disease varies by cohort and gender is unclear despite the increasing importance of education for health, especially among women. Informed by a life-course perspective, this study aims to further contextualize the link between education and heart disease in time and across groups. Using data from the Health and Retirement Study, a nationally representative panel survey of adults over the age of 50, we examine how patterns in intergenerational education are associated with heart disease prevalence (n=31,141) and incidence from 1998 to 2018 (person-year observations=253,521) and whether these patterns vary by gender and cohort. Overall, individuals characterized by stable-high education (i.e., parent and respondent have at least some college education) consistently had the lowest prevalence and incidence of heart conditions, whereas those with low adult education, regardless of parent’s education, had the highest risk of heart conditions. Findings also indicate that the effects of intergenerational education patterns on adult heart disease were most pronounced for women and the younger cohorts compared to men and older cohorts, respectively. For example, women and the youngest cohort (born 1954-1967) with upward and stably high intergenerational education had the most favorable heart disease outcomes, whereas the same groups with downward intergenerational education experienced the highest heart disease prevalence. Our findings demonstrate the importance of contextualizing how intergenerational educational attainment influences later-life heart conditions by considering variations by gender and cohort.

